# A110 INCIDENCE AND PREDICTORS OF ASYMPTOMATIC ABNORMALITIES IN BIOCHEMICAL AND RADIOLOGIC PANCREATIC MARKERS FOLLOWING UNCOMPLICATED ERCP

**DOI:** 10.1093/jcag/gwac036.110

**Published:** 2023-03-07

**Authors:** S Samnani, M Chau, Y Ruan, N Forbes

**Affiliations:** University of Calgary, Calgary, Canada

## Abstract

**Background:**

Post-endoscopic retrograde cholangiopancreatography (ERCP) pancreatitis (PEP) is common. Its diagnosis relies on characteristic abdominal pain in addition to biochemical and/or radiographic evidence of pancreatic inflammation.

**Purpose:**

Little is known regarding the frequency with which asymptomatic alterations in biochemical and/or imaging parameters occur following uncomplicated ERCP. We sought to assess the incidence and predictors of such alterations following uncomplicated ERCP.

**Method:**

This study was an analysis of a prospectively maintained ERCP registry. All inpatients ³ 18 years old who underwent ERCP between 2018/09/01 and 2022/02/28 were identified. Patients with acute pancreatitis or abdominal pain following ERCP were excluded, as were patients with lipase levels ≥3x the upper limit of normal (ULN) within 7 days preceding ERCP. Primary outcomes were (1) asymptomatic lipase elevation within 48 hours of uncomplicated ERCP or (2) asymptomatic cross-sectional imaging findings of pancreatic inflammation within 14 days of ERCP.Descriptive statistics were presented as means with accompanying standard deviations (SD) and percentages by lipase categories and PEP, or by imaging categories. Multiple logistic regression was used to examine the associations of exposure variables with PEP or imaging findings.

**Result(s):**

A total of 646 patients were analyzed in the biochemical cohort, and 187 patients were analyzed in the radiologic cohort. In the biochemical cohort, 478 patients (74.0%) had no elevations in pancreatic enzymes, while 81 (12.5%) had elevations up to 2x ULN, 26 (4.0%) had elevations between 2-3x ULN, and 61 (9.4%) had elevations >3x ULN. In the radiologic cohort, 148 (79.1%) had no abnormalities on cross-sectional imaging within 14 days of ERCP, while 39 (20.9%) had one or more imaging finding typically associated with acute pancreatitis. Among these, 22 (11.8%) had peri-pancreatic fluid collections and 2.1-9.6% of patients had pancreatic findings that included edema, enlargement, inflammation, or fat-stranding. On multivariable analysis, predictors of lipasemia >3x ULN included balloon sphincteroplasty (odds ratio, OR, 2.29, 95% confidence intervals, CI, 1.08 to 4.85) and the placement of a common bile duct stent (OR 4.19, 95% CI 1.37 to 12.77), whereas cannulation of the pancreatic duct or performance of a pancreatogram were not significantly associated (OR 0.75, 95% CI 0.24 to 2.35 and OR 1.07, 95% CI 0.18 to 6.26, respectively).

**Conclusion(s):**

Over 25% of patients will have asymptomatic elevations in pancreatic enzymes following ERCP, while over 20% will have asymptomatic cross-sectional imaging findings suggestive of pancreatic inflammation. Clinical symptoms should guide post-ERCP care rather than biochemical or imaging parameters.

**Please acknowledge all funding agencies by checking the applicable boxes below:**

None

**Disclosure of Interest:**

None Declared

